# Hydration and beyond: neuropeptides as mediators of hydromineral balance, anxiety and stress-responsiveness

**DOI:** 10.3389/fnsys.2015.00046

**Published:** 2015-03-31

**Authors:** Justin A. Smith, Dipanwita Pati, Lei Wang, Annette D. de Kloet, Charles J. Frazier, Eric G. Krause

**Affiliations:** ^1^Laboratory of Dr. Eric Krause, Department of Pharmacodynamics, College of Pharmacy, University of FloridaGainesville, FL, USA; ^2^Laboratory of Dr. Charles Frazier, Department of Pharmacodynamics, College of Pharmacy, University of FloridaGainesville, FL, USA; ^3^Laboratory of Dr. Colin Sumners, Department of Physiology and Functional Genomics, College of Medicine, University of FloridaGainesville, FL, USA

**Keywords:** anxiety, stress, oxytocin, angiotensin, hypernatremia, post-traumatic stress disorder, hypothalamic-pituitary-adrenal axis, paraventricular nucleus

## Abstract

Challenges to body fluid homeostasis can have a profound impact on hypothalamic regulation of stress responsiveness. Deficiencies in blood volume or sodium concentration leads to the generation of neural and humoral signals relayed through the hindbrain and circumventricular organs that apprise the paraventricular nucleus of the hypothalamus (PVH) of hydromineral imbalance. Collectively, these neural and humoral signals converge onto PVH neurons, including those that express corticotrophin-releasing factor (CRF), oxytocin (OT), and vasopressin, to influence their activity and initiate compensatory responses that alleviate hydromineral imbalance. Interestingly, following exposure to perceived threats to homeostasis, select limbic brain regions mediate behavioral and physiological responses to psychogenic stressors, in part, by influencing activation of the same PVH neurons that are known to maintain body fluid homeostasis. Here, we review past and present research examining interactions between hypothalamic circuits regulating body fluid homeostasis and those mediating behavioral and physiological responses to psychogenic stress.

## Introduction

Research focused on developing a comprehensive understanding of circuits that mediate anxiety has been a priority during the past several decades (Griebel and Holmes, 2013). However, lack of therapeutics for anxiety disorders is largely not indicative of this effort (Dias et al., 2013; Stewart and Kalueff, 2014). Although recent advancements in technology are fueling new and promising investigations (Lammel et al., 2014), isolating a treatable anxiolytic pathway in the brain has been elusive. This may be, at least in part, due to the complexity of treating psychiatric disorders in general as, unlike many systemic disorders, the inaccessibility of the brain and the intricacies of its neuronal networks present formidable obstacles to the clear understanding of their origin and progression (for expansion on this topic see Deisseroth, 2014). Thus, novel creative approaches to isolating specific circuits as well as a better understanding of how they dysfunction in anxiety disorders are vital public health concerns. To this end, re-evaluation of the well-characterized renin-angiotensin-aldosterone system (RAAS) and the neuroendocrine systems involving arginine vasopressin (AVP) and oxytocin (OT) is contributing to new mechanistic insight into perhaps underappreciated influences on psychopathology—especially when these systems are considered as complementary homeostatic pathways that impact the appropriateness and timing of responses to psychogenic stress.

Mood disorders are often accompanied by dysregulation of the hypothalamic-pituitary-adrenal (HPA) axis (Arborelius et al., 1999; Jacobson, 2014) which normally serves to mobilize energy reserves through glucocorticoid release in cooperation with the autonomic nervous system (Ulrich-Lai and Herman, 2009). The efficiency of the stress response is governed by (1) the ability to successfully integrate multiple lines of central and peripheral information to reflect a response (Ziegler and Herman, 2002) and (2) the behavioral adaptation and glucocorticoid feedback mechanisms responsible for effecting a timely resolution (Keller-Wood and Dallman, 1984; Watts, 2005). Compelling evidence suggests that systemic and central mechanisms mediating body fluid homeostasis substantially impact both of these parameters.

The physiological stressors of hypotension and hypovolemia stimulate renin release to catalyze angiotensin-II (Ang-II) formation and aldosterone (ALDO) availability (Fitzsimons, 1998), as well as AVP release (Yao et al., 2011). These hormones promote fluid retention to stabilize blood volume and pressure (Fitzsimons, 1998), but also act in the brain to modulate behavior and potentiate stress responsiveness (Krause et al., 2011b) through direct and indirect action in brain regions that innervate the paraventricular nucleus of the hypothalamus (PVH; Englemann et al., 2000; Albrecht, 2010). Low plasma sodium is alleviated by eliminating excess water through diuresis and AVP inhibition (Wang et al., 2013) as well as by increasing salt intake and reducing salt excretion. Hypernatremia increases the pituitary release of AVP and OT to cause renal water reabsorption and salt excretion (Noda and Sakuta, 2013), but the central release of these peptides is known to affect distant forebrain and hindbrain targets (Antunes-Rodrigues et al., 2013) that also influence anxiety (Knobloch et al., 2012; Neumann and Landgraf, 2012; Benarroch, 2013; Frazier et al., 2013). Together these signals represent a constantly fluctuating background upon which the stress response may be potentiated or inhibited depending on the magnitude, polarity, and duration of an osmotic challenge.

The neuroanatomical pathways that govern stress and mood are ongoing subjects of investigation. While many studies have focused on either the RAAS or neuropeptide influences on stress responsiveness separately, a cohesive picture of the specific central pathways that are modulated on a circuit, cellular, and molecular basis is lacking. The brain regions and circuits that govern the habituation, sensitization, and adaptation of the HPA axis that occur during chronic stress have been well-characterized (Flak et al., 2009; Herman, 2013) but whether these circuits and processes are influenced by hydromineral imbalance have only been tenuously addressed. These particular aspects of the stress response are vulnerable to deficiencies or excessive activation of osmoregulatory mechanisms and are directly related to the dysregulation of corticotrophin releasing factor (CRF) and glucocorticoid regulation that are hallmarks of stress-related disorders (de Kloet et al., 2005). Here we address these outstanding issues through a review of the literature and explore the scope of influence and evidence supporting an osmotic component which may heavily influence the function of hypothalamic stress-responsive circuits.

## The RAAS and Stress Responsiveness

The RAAS plays a major role in the regulation of systemic blood pressure, urinary sodium excretion, and renal hemodynamics. In experimental models, stimuli such as pharmacologically induced vasodilation (Stocker et al., 2000), hypovolemia induced by polyethylene glycol administration (Stricker and Verbalis, 1986), or hemorrhage (Yamaguchi and Hama, 2011) are potent activators of the RAAS and elicit thirst. Furthermore, volume depletion associated with sodium loss provokes a rise in Ang-II and ALDO levels (Badauê-Passos et al., 2007). These conditions will be briefly addressed followed by an account of their relevance to the potentiation of stress responsiveness. Finally, experimental reports that demonstrate how increased RAAS activity is related to increased stress-responsiveness and heightened anxiety will be presented.

### Activation of the RAAS

Blood pressure is intrinsically bound to hydromineral balance and deficiency compels several homeostatic mechanisms to action. Carotid sinus and aortic baroreceptors decrease tonic firing frequency in response to a drop in pressure, activating neurons in the nucleus of the solitary tract (NTS) that increase sympathetic outflow while decreasing parasympathetic tone (Fisher and Paton, 2012). If the drop in pressure is severe, afferent signals from the NTS and forebrain induce AVP release which acts on vasopressin type 1 receptors of the vasculature (Aoyagi et al., 2009) and vasopressin type 2 receptors in the kidney (Sampey et al., 1999) to promote vasoconstriction and water reabsorption, respectively. Cardiopulmonary baroreceptors also activate NTS neurons that increase sympathetic nervous system activity. These neurons cause rostroventrolateral medulla and intermediolateral cell column efferents to release norepinephrine onto β1 receptors of the juxtaglomerular apparatus, thereby increasing renin release and plasma renin activity (PRA) to promote the synthesis of Ang-II (Hainsworth, 2014). Additionally, reduced perfusion pressure is sensed by baroreceptors in the renal vasculature to increase PRA via prostaglandin signaling (Schweda and Kurtz, 2011). Prostaglandin-mediated renin release is furthermore induced by a reduction of NaCl circulation in the renal medulla (Schweda and Kurtz, 2011). Thus, reduced extracellular fluid volume is monitored by the systemic vasculature and kidneys to initiate activation of the RAAS as well as hypothalamic nuclei controlling endocrine release and autonomic balance.

Increased circulating Ang-II affects both systemic and centrally-mediated compensatory measures. Ang-II binding to angiotensin type 1 (AT1) receptors is a potent vasoconstrictive stimulus (Fitzsimons, 1998), and activation of AT1 receptors expressed on the efferent arteriole of the kidney increases glomerular filtration rate (Kobori et al., 2013) during instances of low blood pressure. Furthermore, elevated levels of Ang-II upregulate the synthesis and release of ALDO in the zona glomerulosa of the adrenal gland (Hattangady et al., 2012). Aldosterone binds to cytosolic mineralocorticoid receptors within renal tubular cells of the cortical collecting ducts, increasing the reabsorption of NaCl and water (Biller et al., 2001). The effects of Ang-II in the periphery restore extracellular fluid volume by conserving water and NaCl, allowing normal function of vital organs. Ang-II accessibility to the brain however, is blocked by the blood-brain barrier, except for in regions that have a specialized blood-brain barrier architecture (McKinley et al., 2003). These regions, termed circumventricular organs (CVO), allow the brain to sample the immediate systemic environment through specialized neurons (Duvernoy and Risold, 2007). In particular, the subfornical organ (SFO) and organum vasculosum of the lamina terminalis (OVLT) are CVOs that express receptors for Ang-II and ALDO (De Luca et al., 2013). Activation of AT1 receptors in the core of the SFO and lateral margins of the OVLT by Ang-II (Sunn et al., 2003) stimulates a neuronal network with nodes in several brain regions. The net result of Ang-II induced activation of this network is the enigmatic behavioral and physiological phenomenon of thirst (Stricker and Sved, 2000), which is largely dependent on CVO afferent projections to the median preoptic nucleus (Hollis et al., 2008), as well as complex regulation of neuropeptide release (McKinley et al., 2004) and sympathetic activation (Stocker et al., 2008) designed to complement systemic function. Moreover, it is now well-established that the brain is capable of producing all the components of the RAAS (Phillips and Sumners, 1998; Gomez-Sanchez et al., 2005), and systemic Ang-II and ALDO elevations may influence the activity of the local brain RAAS to modulate limbic and neuroendocrine systems.

In regards to ALDO, its central actions are not restricted by the blood-brain barrier, but instead, are limited by competition with glucocorticoids for binding sites (Geerling and Loewy, 2009). Because ALDO circulates at concentrations ~1000 fold less than glucocorticoids (Bledsoe et al., 2005), most mineralocorticoid receptors in the brain are not activated by ALDO. Exceptions include the PVH (Chen et al., 2014); amygdala and locus coeruleus (Robson et al., 1998); and NTS (Geerling and Loewy, 2006), where specialized subsets of cells express both mineralocorticoid receptors and 11β-HSD, making these areas susceptible to physiological states that increase circulating Ang-II and ALDO.

In summary, the RAAS is potently activated by hypotension and reduced extracellular fluid volume and tonicity. Through multiple sensory mechanisms, body fluid homeostasis is maintained through cardiovascular and renal signals that converge on the hypothalamus through CVO and hindbrain inputs, which in turn, are reciprocally connected to limbic brain regions controlling mood and affect. Thus, brain regions controlling mood and affect promote cardiovascular and endocrine responses to stress through hypothalamic and hindbrain nuclei that are also sensitive to RAAS activation. Consequently, RAAS sensitive neuronal networks are positioned to influence cardiovascular and endocrine responses to hydromineral imbalance as well as psychological stressors.

### Potentiation of the Stress Response During Increased RAAS Activity

Stressors can be classified into the two broad categories of physiological or psychological. Physiological stressors include those that threaten body fluid homeostasis, and activation of the RAAS is an example of the body’s attempt to cope through endocrine, autonomic, and behavioral adjustments. Stressors that are psychogenic in nature influence anxiety level, sympathetic tone, and the HPA axis through anticipatory, memory, and fear-related systems relayed to the hypothalamus via limbic brain regions (Jankord and Herman, 2008). Although often unnecessary to the welfare of the individual, coping mechanisms that respond to psychogenic stressors can include RAAS activation (Krause et al., 2011b). Both types of stressors have the potential to activate the HPA axis via stimulation of PVH parvocellular CRF neurons that project to the median eminence. The release of CRF into the primary capillary plexus of the hypothalamo-hypophyseal portal vasculature induces anterior pituitary adrenocorticotrophin hormone (ACTH) release. The binding of ACTH to the zona fasciculata layer of the adrenal gland causes glucocorticoid synthesis and release into the systemic circulation that bind to GRs throughout the body to mobilize energy reserves and down-regulate processes not essential to the stress response. Negative feedback of glucocorticoid occurs at several levels including the pituitary and PVH to bring about termination of HPA axis activity.

Exposure to psychogenic stress activates the RAAS in the absence of threats to hydromineral balance to increase central and systemic Ang-II (Yang et al., 1993) as well as the expression of AT1 receptors in the brain (Aguilera et al., 1995a). Stress-induced up-regulation of AT1 receptor mRNA occurs in the SFO, anterior pituitary, zona glomerulosa and medulla of the adrenal gland (Leong et al., 2002), and on CRF cells in the PVH (Aguilera et al., 1995b). Similarly, administration of dexamethasone, a GR agonist, significantly increases AT1 receptor binding in the PVH and SFO (Shelat et al., 1999). Taken together, these results suggests that glucocorticoids derived from stress-induced HPA axis activation may feed forward to increase AT1 receptor expression to alter sensitivity to centrally released Ang-II. Consistent with this notion, pretreatment with dexamethasone augments drinking stimulated by central administration of Ang-II (Ganesan and Sumners, 1989) and elevated glucocorticoids accompanied by increased Ang-II release in the PVH reduces 11β-HSD effectiveness resulting in increased pre-autonomic stimulation (Gabor and Leenen, 2012) and potentiation of sympathetic activity (de Kloet et al., 2005). Thus, induction of the HPA axis may interact with an activated RAAS to sensitize subsequent behavioral and autonomic responses to hydromineral imbalance; however, central angiotensinergic circuits activated by subsequent exposure to a psychogenic stressor may also be primed for heightened stimulation.

### Pathways Mediating RAAS-Potentiated Stress-Response

The RAAS has gained considerable attention over the past decade as a potential therapeutic target for treating stress-related disorders, anxiety, depression, alcoholism, cognitive impairment as well as neuroinflammatory and neurodegenerative diseases (Saavedra and Pavel, 2005; Albrecht, 2010; Saavedra, 2012; Anil Kumar et al., 2014). Angiotensin receptor blockers administered either peripherally or centrally, improve mood and cognition as well as attenuate HPA axis and sympathetic responses to stressors having strong psychogenic components (Saavedra and Pavel, 2005; Albrecht, 2010; Saavedra, 2012; Anil Kumar et al., 2014). While the specific circuits that angiotensin receptor antagonists modulate to achieve these affects are not fully understood, increased circulating Ang-II is known to influence hypothalamic function through various pathways including those originating in CVOs and regions governing mood and affect.

#### Lamina Terminalis to Paraventricular Nucleus

The SFO and OVLT, together with the median preoptic nucleus represent key regulatory areas that both share connectivity with one another and project to the hypothalamus. As previously discussed, the SFO and OVLT are unique in their ability to sample the constituents of the systemic circulation and promote activation of circuits to preserve homeostasis. Activation has been shown to alter the balance between excitatory and inhibitory projections to the PVH and SON which strongly influences the activity of the HPA axis and behavior.

Excitation of PVH-mediated neuroendocrine and autonomic regulation can be inhibited with the angiotensin-receptor blocker losartan (Bains and Ferguson, 1993; Li and Ferguson, 1993). These studies also support the role of Ang-II acting as a neurotransmitter specifically in an SFO to PVH circuit. More recent investigation into the functionality of the angiotensinergic SFO to PVH circuit found that while Ang-II in the SFO significantly increased renal sympathetic nerve activity and heart rate, inhibition of these effects could only be accomplished by PVH pretreatment with glutamate (GLUT) receptor antagonist and not losartan (Llewellyn et al., 2012). Together, the results of these studies suggest that SFO to PVH neurotransmission that is initiated by Ang-II binding in the SFO may be either glutamatergic or angiotensinergic depending on the stimulus. Indeed, on a cellular basis Ang-II activation of AT1 receptors stimulates water intake or salt intake through separate intracellular signaling pathways (Daniels et al., 2009).

Hypovolemia-induced activation of the SFO and OVLT influence local γ-amino butyric acid (GABA) and GLUT circuits between these nuclei and the median preoptic nucleus (Kolaj et al., 2004). The activity of GABA neurons projecting from the SFO and OVLT to the median preoptic nucleus is driven by hindbrain norepinephrine afferents to these CVOs through activation of α-1 and α-2 adrenoreceptors (Kolaj and Renaud, 2007). Antagonism of GABA_A_ receptors to model this inhibitory influence resulted in suppression of hypotension-induced AVP release (Yamaguchi and Hama, 2011) suggesting that local inhibitory circuits originating in the SFO and OVLT regulate the corresponding PVH responses to stimuli that elevate systemic Ang-II. A similar mechanism was modeled with an angiotensinergic OVLT to SON circuit. Bourque et al. found that glutamatergic osmosensitive neurons increased the output of AVP neurons, but decreased the output of OT neurons in the presence of Ang-II (Stachniak et al., 2014). Thus, while Ang-II signaling in the periphery initiates compensatory circuits, Ang-II acting as a neuromodulator is capable of tuning appropriate GABAergic and glutamatergic inputs to the hypothalamus.

**Figure 1:**
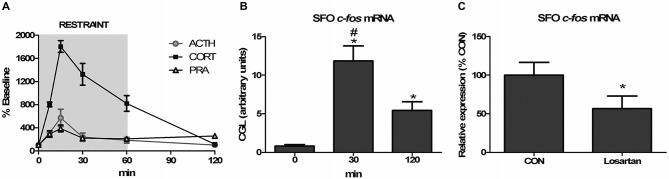
**Angiotensin receptor blockade attenuates restraint-induced c-fos expression in the subfornical organ (SFO). (A)** Sixty-minute restraint elicits elevations in adrenocorticotropic hormone (ACTH), corticosterone (CORT), and plasma renin activity (PRA) with similar peaks at 15 min (*n* = 8 per group and time point). **(B)** Restraint significantly increases c-fos mRNA in the SFO asterisk (*), different than 0 min; pound sign (#), different than 120 min; *,#*p* < 0.05, *n* = 8 per group). **(C)** Restraint-induced c-fos mRNA expression in the SFO is attenuated by systemic pretreatment with the AT1 receptor antagonist losartan (*p < 0.05, *n* = 6 per group). Reprinted with permission from Krause et al. (2011b).

**Figure 2:**
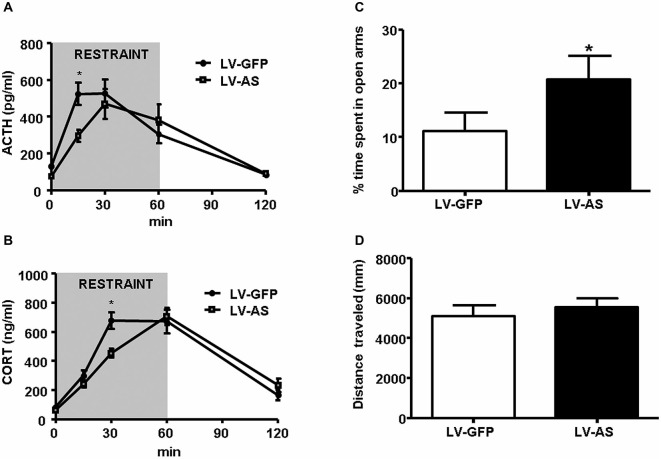
**Inhibition of angiotensin type 1 (AT1) receptor transcription in the SFO diminishes stress-responsiveness and anxiety-like behavior. (A,B)** Administration of a lentiviral antisense (LV-AS) targeting an AT1 receptor coding region vs. lentiviral green fluorescent protein expressing control (LV-GFP). Injection into the SFO significantly decreases (*p < 0.05) the release of ACTH **(A)** and CORT **(B)** during restraint (ACTH LV-GFP, *n* = 12; ACTH LV-AS, *n* = 12; CORT LV-GFP, *n* = 16; CORT LV-AS, *n* = 15). **(C,D)** Delivery of LV-AS into the SFO significantly increases (*p < 0.05) the amount of time spent in the open arms of the elevated plus maze (EPM) but has no effect on total distance traveled (*n* = 12 per group). Reprinted with permission from Krause et al. (2011b).

The convergence of signaling through multiple osmosensitive circuits results in behavioral changes and altered hypothalamic regulation of stress-responsiveness. The behavioral outcome of optogenetic stimulation of SFO afferents were recently demonstrated in a dramatic way as excitatory projections robustly increased thirst while inhibitory projections blocked drinking behavior (Oka et al., 2015). Thirst as a result of hypovolemia is associated with increased circulating Ang-II, and activation of the pathways discussed above. Moreover, acute hypotension increased Ang-II stimulated thirst, ACTH and glucocorticoid release, and these increases were attenuated by knocking-down AT1 receptors in the SFO, but not in the OVLT (Krause et al., 2008). In a subsequent study, it was determined that restraint-induced elevations in PRA and SFO activation occurred with a time course commensurate with those of ACTH and glucocorticoids (Krause et al., 2011b; See Figure 1). Interrupting AT1 receptor expression in the SFO both suppressed the HPA response and reduced anxiety-like behavior (Krause et al., 2011b; See Figure 2). Furthermore, anterograde tracer injection revealed SFO neurons making appositions onto PVH neurons that were also activated by restraint (Krause et al., 2011b), supporting the hypothesis that systemic Ang-II acts as a stress hormone and anxiogenic agent through an SFO-PVH connection.

#### Dorsal Raphe to Lamina Terminalis

Blocking Ang-II receptors improves mood and affect (Dimsdale, 1992) and there is anatomical and functional evidence that these effects may be regulated, in part, by serotonin (5-HT). A bi-directional connection exists between the SFO and the dorsal raphe nucleus (DRN), the brain’s primary source of serotonin (Lind, 1986). 5-HT released in the SFO has both excitatory and inhibitory effects, and influences cells that are also sensitive to Ang-II (Scrogin et al., 1998). Functionally, hypovolemia-induced drinking is attenuated by i.c.v. pretreatment with a 5-HT1C/5-HT2 agonist (Reis et al., 1992) and 5-HT1D agonist injected into the 3rd ventricle reduces water intake in dehydrated rats (De Castro-e-Silva et al., 1997) suggesting that serotonergic innervation of the lamina terminalis is capable of disrupting the dipsogenic effects of Ang-II. Caudal DRN neurons projecting to the lamina terminalis are activated by urocortin-2 (Hale et al., 2010), a stress and anxiety related neuropeptide that binds to the CRF type-2 receptor and is generally thought to produce anxiolytic effects. The beneficial mood-enhancing effects of this pathway may be attenuated by activation of the systemic RAAS, as release of 5-HT in the SFO is reduced in the presence of Ang-II (Tanaka et al., 2003). Collectively, these results suggest a complex interaction between Ang-II and 5-HT in the SFO that may influence both water intake and anxiety levels, and further research is needed to fully understand the circuits by which anxiolysis is produced by blocking the effects of Ang-II.

#### Subfornical Organ to Bed Nucleus of the Stria Terminalis (BNST) and Lateral Hypothalamus

The amygdala and bed nucleus of the stria terminalis (BNST) are brain regions known to regulate the behavioral and physiological responses to conditioned fear (Walker et al., 2003). Ressler et al. recently found that systemic administration of the AT1 receptor antagonist, losartan, reduced Pavlovian fear-conditioning and decreased AT1 mRNA in the amygdala and BNST (Marvar et al., 2014). Systemically delivered losartan is impermeable to the blood-brain-barrier (Bui et al., 1992); however, peripheral administration of losartan does antagonize AT1 receptors residing in CVOs and suppresses stress-induced activation of the SFO (Krause et al., 2011b). Similar to the PVH, the BNST receives strong direct projections from SFO neurons and it is possible that stress-induced elevations in blood-borne Ang-II activates AT1 receptors expressed on SFO neurons to influence neuronal activation within brain nuclei mediating fear and anxiety. In this regard, systemic administration of losartan decreased neuronal activation, as indicated by c-fos mRNA, in the BNST subsequent to extinction of conditioned fear (Marvar et al., 2014). The SFO is also well-positioned to influence the cardiovascular response to conditioned fear. The amygdala sends direct projections to the lateral hypothalamus and this connection is implicated in the cardiovascular responses to emotionally relevant stimuli (Smith et al., 1990). The lateral hypothalamus sends angiotensinergic projections to the SFO (Lind et al., 1985) and pressor responses elicited by activation of amygdalar connections to the lateral hypothalamus can be attenuated by microinjections of angiotensin receptor antagonist into the SFO (Ku and Li, 2003). Collectively, these results suggest that AT1 receptor signaling within CVOs may influence neuronal activation within limbic and hypothalamic nuclei, which in turn, alters behavioral and physiological responses to psychogenic stress.

#### Alternative Pathway of Angiotensin Signaling

A novel carboxypeptidase which shares 40% identity and 61% similarity with angiotensin-converting enzyme (ACE) was discovered in 2000 (Tipnis et al., 2000). This ACE homolog was named angiotensin-converting enzyme 2 (ACE2) based on its ability to cleave Ang-II to produce angiotensin-(1-7) (Ang-(1-7)) (Donoghue et al., 2000; Tipnis et al., 2000). Ang-(1-7) counter-regulates many of the effects of the classic RAAS through activation of the Mas receptor (Iwai and Horiuchi, 2009). Interestingly, the components of the ACE2/Ang-(1-7)/Mas axis are expressed in brain regions which regulate hydromineral balance and stress responsiveness including the SFO, limbic system, PVH, NTS, and ventrolateral medulla (Becker et al., 2007; Doobay et al., 2007). Thus, the counter-regulatory limb of the RAAS is anatomically well-placed to control hydromineral balance as well as stress responsiveness. For example, ACE2 overexpression within the SFO blunts the drinking and pressor responses elicited by centrally administered Ang-II (Feng et al., 2008). The same study found that ACE2 overexpression down-regulated AT1 receptor expression in the SFO (Feng et al., 2008). AT1 receptor expressing neurons in the SFO are thought to promote anxiety and potentiate stress responsiveness through connections to the PVH (Krause et al., 2011b). Therefore, it is possible that the ACE2/Ang-(1-7)/Mas axis may have stress dampening and anxiolytic effects through actions on the SFO and other stress responsive brain nuclei. In this regard, rodents given Ang-(1-7) i.c.v. demonstrate reduced anxiety-like behavior (Bild and Ciobica, 2013) and attenuated cardiovascular responsiveness to psychogenic stress (Martins Lima et al., 2013). Preliminary results from our laboratory also suggest that manipulations that up-regulate ACE2/Ang-(1-7)/Mas axis activity are anxiolytic and stress dampening. Specifically, mice genetically engineered to overexpress ACE2 exhibit suppressed anxiety-like behavior in the elevated plus maze (EPM) and an attenuated glucocorticoid response to restraint and these effects are eliminated by central administration of a Mas receptor antagonist (Wang, 2013). Currently, ACE2 is in clinical trials for the treatment of cardiovascular and metabolic disorders; however, the therapeutic utility of targeting the ACE2/Ang-(1-7) axis for the alleviation of mental health disorders may be only beginning to be realized.

### Summary

Real threats to body fluid homeostasis and perceived threats derived from psychogenic origins activate the RAAS. Blood-borne Ang-II stimulates AT1 receptors expressed on neurons residing in CVOs and these neurons have direct connections to hypothalamic and limbic nuclei controlling physiological and behavioral responses to systemic and psychogenic stressors. In addition, AT1 receptors are present on neurons within the confines of the blood-brain barrier and their expression is regulated by hydration state and stress exposure. These AT1 receptors likely bind brain-derived Ang-II and influence the function of neurons mediating hydromineral balance and cardiovascular function as well as mood and affect. Looking forward, in addition to AT1 receptors, it may be that brain renin, angiotensinogen, or components of the ACE2/Ang-(1-7)/Mas axis will need to be considered to fully realize the therapeutic potential of targeting neuronal circuits that are influenced by the RAAS.

## Oxytocin, Vasopressin, and Stress Responsiveness

The previous section discussed the neuronal circuits by which the RAAS maintains body fluid homeostasis but also influences the behavioral and physiological responses to psychogenic stress. As mentioned, the major stimulus for RAAS activation is sodium depletion or hyponatremia. However, excess body sodium or hypernatremia is a potent inhibitor of the RAAS and creates a distinct neural and humoral milieu that alleviates elevations in body sodium levels. The following section reviews the hypothalamic circuits that maintain hydromineral balance when challenged with hypernatremia and discusses their overlap and interactions with those that control stress responsiveness and anxiety.

### Neuropeptide Responses to Alleviate Elevated Plasma Tonicity

The acute elevation of plasma tonicity that occurs following hypertonic saline administration creates an osmotic gradient which moves water from the intracellular fluid compartment to the extracellular fluid, which in turn, increases blood volume and pressure (Caeiro and Vivas, 2008). The loss of water from the intracellular fluid compartment causes cell shrinkage that is thought to activate osmoreceptors located on the membrane of specialized osmosensitive cells. Research investigating osmoreceptor function support the notion that changes in membrane tension elicit conformational changes in the osmoreceptor that lead to increased membrane ion permeability, thereby transducing the change in osmolality into a signaling mechanism that can affect other cells (Voisin and Bourque, 2002; Bourque, 2008). Osmosensitive cells are located in strategic areas including the throat (Kuramochi and Kobayashi, 2000), intestines (Dooley and Valenzuela, 1984), hepatic portal vein (Baertschi and Vallet, 1981) and CVOs in the brain (McKinley et al., 1999). In regards to CVOs, the OVLT conveys information regarding a change in osmolality directly to AVP and OT producing cells in the SON and PVH (Johnson and Gross, 1993). The release of AVP into the systemic circulation counters the increased plasma osmolality by promoting renal water retention. In rodents, the elevations in systemic OT that follows hypernatremia promotes the inhibition of renin release and stimulates renal sodium excretion (Sjöquist et al., 1999). Thus, increased plasma osmolality due to excess body sodium elicits activation of osmosensitive cells causing subsequent release of neuropeptides into the systemic circulation that alter the renal handling of sodium and water to alleviate hypernatremia.

### Hypernatremia-Induced Dampening of Stress Responsiveness Through Hypothalamic Circuits

**Figure 3:**
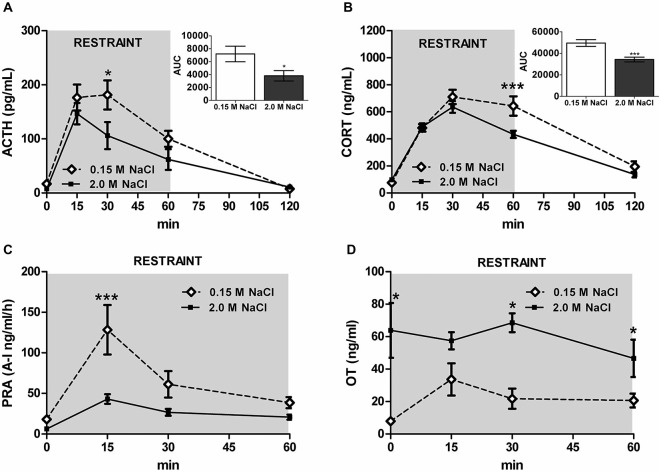
**Acute hypernatremia reduces stress-responsiveness and PRA, but increases plasma oxytocin (OT). (A)** Restraint elevates ACTH; however, rats treated with 2.0 M NaCl have a blunted response compared with controls. **(B)** Similarly, restraint increases CORT, but rats treated with 2.0 M NaCl have decreased CORT relative to controls. **(C)** Restraint significantly increases PRA at 15 min for controls, but a significant increase was not detected in rats given 2.0 M NaCl. **(D)** Relative to controls, rats injected with 2.0 M NaCl have increased systemic OT before and 30 and 60 min after the onset of restraint. ACTH/CORT, *n* = 12 per condition; PRA/OT, *n* = 5–6 per time point. ***p < 0.01; *p < 0.05. Error bars indicate SEM. Reprinted with permission from Krause et al. (2011a).

**Figure 4:**
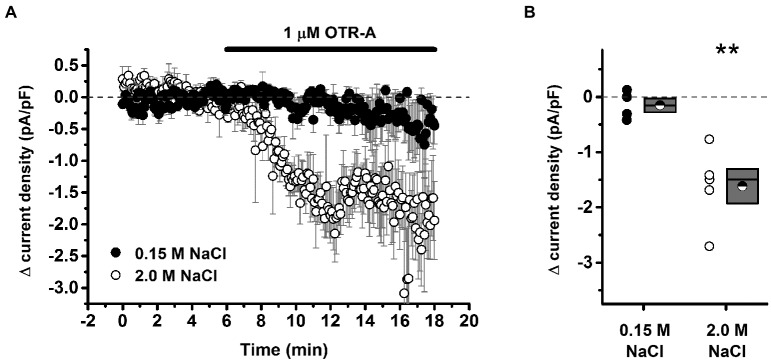
**Acute hypernatremia is associated with an oxytocin-dependent inhibitory tone on putative stress-responsive cells of the paraventricular nucleus. (A)** Illustration of the change in current density as observed during bath application of 1 μM OTR-A, an oxytocin receptor antagonist. A decrease in current density is indicative of removal of an inhibitory oxytocinergic tone but was observed only in slices taken from hypernatremic rats. **(B)** Summary data for all cells that met criteria for this experiment. Box plots for each group illustrate the mean (half-filled circle), the median (horizontal line with in the box), and the SEM (top and bottom edges of the box) are shown. The double asterisks over the 2.0 M NaCl group indicate that the mean change in current density observed in this group is significantly greater than 0 (*p* = 0.007) and also significantly greater than the change observed in the 0.15 M NaCl group (*p* = 0.006). By contrast, the mean change observed in the 0.15 M NaCl group was not significantly different from 0 (*p* = 0.32). **p < 0.01. Reprinted with permission from Frazier et al. (2013).

**Figure 5:**
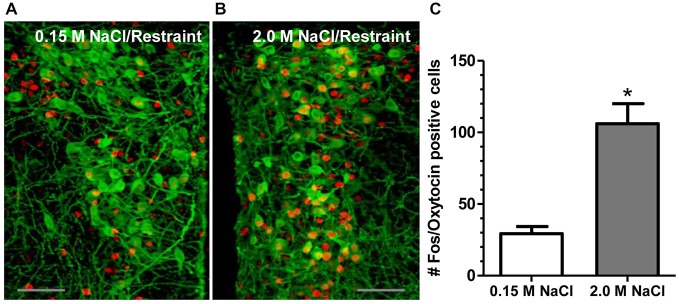
**2.0 M NaCl administration and restraint increases activation of OT-producing cells in the paraventricular nucleus of the hypothalamus (PVH). (A)** Representative photomicrograph of a unilateral coronal section depicting Fos induction (red nuclei) in OT (green cell bodies) containing neurons following 0.15 M NaCl and restraint. **(B)** Representative photomicrograph of a unilateral coronal section depicting Fos induction in OT neurons following 2.0 M NaCl and restraint. **(C)** The group mean for Fos induction in OT-producing cells was significantly more for mice subjected to 2.0 M NaCl injection and restraint relative to control. *p < 0.05 Scale bars = 50 μm. Reprinted with permission from Smith et al. (2014).

**Figure 6:**
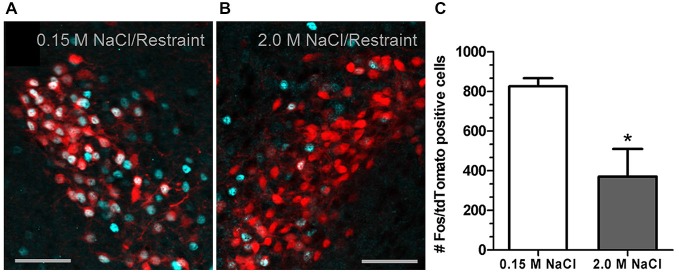
**2.0 M NaCl injection attenuates restraint-induced activation of corticotrophin-releasing factor (CRF) neurons in a transgenic reporter mouse. (A)** Representative photomicrograph of a unilateral coronal section through the PVH depicting Fos induction (cyan nuclei) in tdTomato (red soma) containing neurons following 0.15 M NaCl and restraint. **(B)** Representative photomicrograph of a unilateral coronal section through the PVH depicting Fos induction in tdTomato containing neurons following 2.0 M NaCl and restraint. **(C)** The group mean for Fos induction in tdTomato containing neurons was significantly less for mice subjected to 2.0 M NaCl injection and restraint relative to control. *p < 0.05 Scale bars = 50 μm. Reprinted with permission from Smith et al. (2014).

Recent investigation into the effects of hypertonic saline injection on humoral, behavioral, and central measures of stress responsiveness in male rats and mice revealed a prominent role for activation of oxytocinergic pathways. Hypertonic (2.0 M) saline injected s.c. followed by 60 min water deprivation resulted in decreased ACTH, glucocorticoid, and PRA during a 60 min restraint period, but significantly increased plasma OT levels (Krause et al., 2011a; See Figure 3). Cardiovascular recordings determined that rats rendered mildly hypernatremic and subsequently exposed to restraint stress had mean arterial pressure and heart-rate variability return to pre-restraint levels faster than normonatremic controls (Krause et al., 2011a). Consistent with previous reports (Pirnik et al., 2004) acute hypernatremia increased Fos induction in AVP and OT expressing neurons in the PVH and SON, an effect that was predictive of increased social interactions but decreased anxiety-like behavior in the elevated plus maze (Krause et al., 2011a). Acute hypernatremia and restraint stress interacted to increase Fos expression in the OVLT, oval capsule of the BNST, ventral lateral septum, and central nucleus of the amygdala (Frazier et al., 2013). However, the increased neuronal activation that occurred subsequent to acute hypernatremia and restraint was specific to these brain regions as the medial pre-frontal cortex, lateral ventral BNST, and dorsomedial hypothalamus showed no significant change in Fos expression (Frazier et al., 2013). Whole cell patch clamp recordings revealed that acute hypernatremia caused inhibition of CRF neurons in the PVH that was dependent on activation of OT receptors (Frazier et al., 2013; Figure 4). Follow-up studies utilized the Cre-lox system to generate a line of CRF reporter mice to investigate the mechanisms underlying the anxiolysis and HPA dampening that accompanies acute hypernatremia (Smith et al., 2014). Acute hypernatremia elicited robust activation of OT neurons in the PVH but attenuated the Fos induction that occurs in CRF neurons subsequent to restraint stress (Smith et al., 2014; See Figures 5, 6), suggesting that the oxytocinergic tone that inhibited CRF neurons in electrophysiological experiments suppresses stress-induced activation of CRF neurons *in vivo*.

#### Local Hypothalamic Circuits Regulated by Dendritic Release of AVP and OT

Our laboratory and others (Huang et al., 2014) are actively pursuing the neuronal mechanism underlying the dampened HPA activation that occurs with acute hypernatremia. Seminal studies conducted by Ludwig et al. discovered that subsequent to the systemic release of AVP and OT that follows peripheral salt-loading, brain levels of these neuropeptides exhibit a robust and sustained increase (Ludwig et al., 1994). The implication is that magnocellular neurons in the SON and PVH release neuropeptides from dendrites and these neuropeptides may act as autocrine or paracrine signals at their site of origin. In this regard, dendritic release of AVP serves as a powerful autocrine signal that modulates firing patterns and activity of magnocellular AVP neurons in the SON and PVH (Ludwig and Leng, 1997; Gouzènes et al., 1998). Recently dendritically released neuropeptide also was found to signal in a paracrine fashion to influence the functional activity of neighboring neurons of a differing neuronal phenotype. Specifically, the Stern laboratory discovered that dendritic release of AVP stimulated neighboring presympathetic neurons in the PVH to drive sympathoexcitation following a hyperosmotic stimulus (Son et al., 2013). Hyperosmotic stimuli also trigger the dendritic release of OT in the hypothalamus and (Ludwig and Leng, 2006; Mabrouk and Kennedy, 2012) OT neurons are in close proximity to CRF neurons that express OT receptor mRNA in the PVH (Dabrowska et al., 2011). Therefore, it is possible that the inhibition of CRF neurons that accompanies acute hypernatremia maybe regulated by dendritically released OT that activates OT receptors expressed on CRF neurons in the PVH.

#### Paraventricular and Supraoptic Nuclei of the Hypothalamus to the Amygdala

Identifying the oxytocinergic circuits underlying the anxiolytic effects of acute hypernatremia may reveal neuronal mechanisms that quell excitation of neurons that facilitate physiological and behavioral manifestations of fear and anxiety. The magnocellular neurons in the PVH and SON are the major source of OT but whether these neurons supplied OT to forebrain nuclei controlling mood and affect was unclear because their axons are known to terminate in the posterior pituitary where they release OT into the systemic circulation. Consequently, endogenously generated OT was hypothesized to activate OT receptors in forebrain nuclei by diffusion of dendritically released peptide (Ludwig and Leng, 2006) or via axonal release from a subset of parvocellular oxytocinergic neurons residing in the PVH that do not project to the pituitary (Swanson and Sawchenko, 1983). These hypotheses about the source of centrally released OT have undergone a paradigm shift as the result of recent research utilizing advanced techniques in neuroanatomical tract-tracing. These elegant studies conducted by Grinevich et al. discovered that OT neurons in the PVH and SON that project to the posterior pituitary also send axon collaterals to forebrain nuclei mediating stress responsiveness and anxiety-like behavior (Knobloch et al., 2012). In other words, oxytocinergic magnocellular neurons in the PVH and SON simultaneously project to the posterior pituitary and the forebrain, presenting the possibility that systemic and central release of OT maybe accomplished by the same neurons. In rodents, magnocellular oxytocinergic neurons in the PVH and SON are robustly excited by elevations in the pNa^+^ (Krause et al., 2011a; Frazier et al., 2013; Smith et al., 2014). Therefore, it is possible that acute hypernatremia is a systemic stimulus that excites hypothalamic osmosensing neurons to promote the release of OT into systemic circulation, and subsequently, stimulates the release of OT from axons terminating in forebrain nuclei known to mediate physiological and behavioral manifestations of fear and anxiety. In support of this idea, ongoing studies suggest that acute hypernatremia creates an inhibitory tone on CRF neurons residing in the central nucleus of the amygdala that is dependent on activation of OT receptors (Krause, 2014). Amygdalar CRF neurons are implicated in the development of anxiety disorders and further research is warranted to determine whether this potential oxytocinergic pathway is a viable therapeutic target.

#### Paraventricular Nucleus to the Bed Nucleus of the Stria Terminalis

In addition to the central nucleus of the amygdala, the extensive mapping of oxytocinergic afferents throughout the rat brain isolated dense innervation of the BNST (Knobloch et al., 2012). The BNST is a complex set of at least a dozen sub-nuclei that mediate the information gathered by higher-order perceptual and memory-based brain regions through an integrating process involving the brain-stem and hypothalamus (Dong et al., 2001; Bota et al., 2012). Because of this pivotal role, the BNST is rich in neurochemical diversity, both locally and in terms of the phenotype of afferent and efferent projections (Dong and Swanson, 2006; Bota et al., 2012). Rainnie et al. used viral anterograde tracing of PVH neurons to reveal that OT immunoreactive fibers terminating in the BNST originate from neurons in the PVH (Dabrowska et al., 2011) and subsequent studies suggest that these cells are osmosensing magnocellular neurons (Knobloch et al., 2012). Acute hypernatremia followed by restraint-stress robustly activated OT neurons in the PVH (Krause et al., 2011a; Smith et al., 2014) as well as neurons residing in the oval capsule portion of the BNST (Frazier et al., 2013). These studies support the hypothesis that elevations in body sodium levels excite magnocellular OT neurons and subsequent psychogenic stress exposure may trigger the release of OT from axons terminating in the BNST, which in turn, activate neurons within this nucleus. The pattern of neuronal activation that occurs after acute hypernatremia and restraint is predictive of decreased anxiety-like behavior and increased social interactions (Krause et al., 2011a). In this regard, Kim et al found that two BNST sub-regions have highly specific roles in mediating separate features of anxiety such as risk-avoidance and respiratory rate (Kim et al., 2013). While this study found that activation of the BNST increased anxiety, activation associated with acute hypernatremia seemed to be related to a decrease in anxiety-like behavior. As mentioned, the BNST is a highly diverse region in terms of cellular capabilities and connectivity that may be the basis for understanding the apparent discrepancies in activation of this region. Ultimately, the circuit level data that has produced valuable insights into the effects of activating cells in these areas will have to be complemented by studies that take into consideration ligand-receptor interactions.

### Summary

An acute osmotic challenge induces anxiolytic and pro-social effects associated with an increase in OT-producing cell activation and a reduction in the HPA and behavioral components of the stress response. As OT cells in the PVH are both osmosensitive and receive projections from osmosensitive areas of the brain, the increase in activation is likely due to the pivotal role of OT in rectifying the fluid imbalance. Through dendritic release and collateralized axonal projections that target the posterior pituitary and limbic brain regions influencing mood and behavior, OT signaling orchestrates the humoral and behavioral responses necessary to maintain hydromineral homeostasis specifically in the context of an acute hyperosmotic stimulus.

## Clinical Studies

Clinical correlates to the presented experimental findings using animal models are abundant. Increased activity of the RAAS is predictive of depression and anxiety, and when medication that blocks or inhibits a specific component of the RAAS is prescribed for hypertension, there has also been improvement in mood and affect. Large scale studies have provided evidence that ALDO levels may be a possible solution to the long-sought after biomarker for affective disorders. Furthermore, direct evidence for the anxiolytic and stress-dampening effects of OT is available although there have been conflicting reports.

### Renin-Angiotensin-Aldosterone System

Interest in the relationship between anxiety or depression and RAAS activity found significant correlations. In a two-year long study involving 1805 participants, researchers found that depressed hypertensive individuals had elevated ALDO levels compared with individuals that were not depressed or those that were not hypertensive (Häfner et al., 2013). Participants that were either only depressed or, surprisingly, only hypertensive did not present with heighted renin or ALDO. In a smaller, but much more specific study carried out while participants were asleep, Murck et al. monitored HPA and RAAS hormones of unmedicated depressed and age-matched control subjects finding that ALDO was significantly higher in depressed individuals (Murck et al., 2003, 2012). Furthermore, more than half of patients with primary aldosteronism surveyed also suffered from an anxiety disorder and these individuals had higher levels of self-reported stress, psychological distress, and a lower level of well-being (Sonino et al., 2006, 2011). As previously discussed, ALDO affects specific areas of the brain in rodent models, including the PVH, NTS, locus coeruleus, and amygdala. The clinical data showing a correlation between elevated ALDO with anxiety and depression suggests that these areas are perhaps viable targets for intervention if the primary cause of hyperaldosteronism cannot be directly addressed.

### Oxytocin

MacDonald and Feifel recently reviewed progress in developing OT-based therapeutics, citing over 30 clinical studies using intranasal administration of OT in normal individuals as well as single and multi-dose trials in those suffering from a brain-based illness (MacDonald and Feifel, 2014). Results in terms of efficacy in reducing anxiety were conflicting; however, some promising anatomical data was identified. For instance, females with a common OT receptor gene variant present with a psychological phenotype that includes excessive worrying, cautiousness, pessimism, shyness, and fearfulness (Wang et al., 2014). This variant is associated with reduced resting state PFC to amygdala connectivity as assessed by fMRI and this circuit’s functional connectivity is improved with OT administration (Dodhia et al., 2014). It may be that more specific routes of altering the central OT system will be necessary in order to tailor OT-based therapies to individual disorders.

A better understanding of the oxytocinergic circuitry may lead to novel therapeutic strategies for treating core symptoms of autism spectrum disorders (ASD). Anecdotal reports and clinical case studies have found that illness accompanied by fever markedly alleviates symptoms of ASD (Sullivan et al., 1980; Cotterill, 1985; Curran et al., 2007). A hallmark of fever is insensible water loss that causes acute hypernatremia or increases in the plasma sodium concentration. Thus, it is plausible that hypernatremia driven increases in central levels of endogenous OT might alleviate symptoms of ASD. Accumulating preclinical and clinical evidence suggests that brain oxytocinergic circuits are valid candidate therapeutic targets for relief of ASD symptoms (Andari et al., 2010; Guastella et al., 2010; Domes et al., 2013; Taurines et al., 2014).

The ability of OT to suppress the activity of PVH CRF neurons seems to be an effect seen in both humans and animal models. As discussed previously, an acute hypernatremic state is associated with increased central release of OT that potentiates an inhibitory OT-dependent tone on CRF neurons of the PVH. In support of this, human males were given intranasal OT in a randomized, placebo-controlled, double-blind experiment to test the effect on exercise-induced salivary cortisol levels finding 24 IU, but not 48 IU OT significantly attenuated cortisol levels compared with placebo (Cardoso et al., 2013). Further research of this caliber is necessary to determine if similar results are obtained with the induction of a psychogenic stressor.

## Closing Remarks

Relatively free, widespread access to potable water and an excess of salt are contemporary additions to most civilized societies, representing only a small fraction of human evolution. This suggests that the hydration-related instincts that for millennia guided behavior, may be as limited in their usefulness to modern man as many misappropriated aspects of the stress response. However, for many non-human species the centrally-mediated mechanisms connecting hydromineral homeostasis with anxiety levels may have developed to improve the likelihood of survival. Activation of the RAAS is associated with physiological states such as sodium and volume depletion, situations which would also have the potential to produce an anxious state. For example, species that do not consume meat have a dietary imperative to locate and consume salt. Freely accessible salt may represent isolated areas that may also be known to predators as a successful hunting area. Thus, the low-sodium state and locating of a salt source may be hazardous and necessitate the increased alertness of heightened anxiety. Furthermore, a mild hypernatremic state is relieved primarily by approaching a water source that may also be frequented by a variety of species implying that the increased sociability and decreased anxiety that accompanies central OT release may be of survival value as well. It may be that these evolutionarily conserved systems as well as the circuits that interact with them may become dysregulated by a variety of genetic and environmental influences.

The understanding of a pathological process obviously begins with a clear understanding of normal physiological function. Indeed, the development of our understanding of the RAAS occupies a large segment of modern medical history which has revolutionized pharmacological treatment for hypertension. Pushing the boundaries of this knowledge has uncovered local tissue-specific systems with RAAS components as well as a counter-regulatory limb that will likely result in novel therapeutic targets. Furthermore, it is clear that increased activation of the RAAS promotes anxiety and potentiates the response to a psychogenic stressor. While existing drugs have been shown to improve these symptoms, it is still unclear exactly how these off-target effects are accomplished. Similarly, OT and AVP have well-characterized endocrine effects in the body, but are just beginning to be thought of as centrally acting mediators of behavior. Together, the mechanisms of salt and water homeostasis represent a toolbox for uncovering what may be fundamental central processes with unrealized potential.

**Figure 7:**
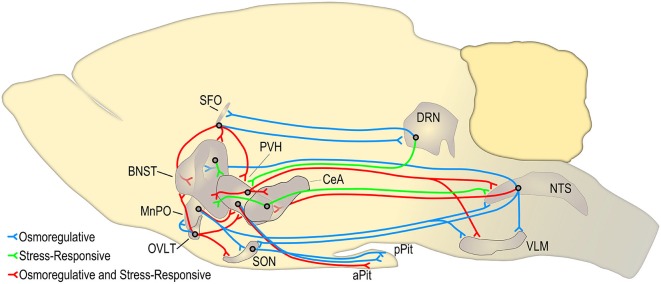
**Circuits adapted in part from Ulrich-Lai and Herman (2009) and Bourque (2008) demonstrating key hypothalamic circuits that mediate the behavioral, endocrine, and autonomic stress-response (shown in red and green)**. Many of these circuits are also activated when the plasma osmolality is elevated above a threshold set point (shown in blue and red). The circuits shown in red represent stress-responsive pathways that may be altered during an osmotic challenge such as hypernatremia. This figure is not exhaustive and does not represent circuits that are necessarily monosynaptic. The borders and relative size of each brain region were determined using Brain Explorer 2.^1^ SFO, subfornical organ; BNST, bed nuclei of the stria terminalis; MnPO, median preoptic nucleus; OVLT, organum vasculosum of the lamina terminalis; PVH, paraventricular nucleus of the hypothalamus; CeA, central nucleus of the amygdala; SON, supraoptic nucleus; DRN, dorsal raphe nucleus; aPit, anterior pituitary; pPit, posterior pituitary; NTS, nucleus of the solitary tract; VLM, ventral lateral medulla.

The primary advantages of studying the central effects of both the RAAS and the neuropeptides OT and AVP is that their systemic roles in normal physiological function have been extensively studied and the routes by which they activate brain circuits are well-understood (See Figure 7). With these rigorously reviewed research findings at hand, it is possible to manipulate the normal homeostatic mechanisms governing hydromineral balance for the purpose of studying the effect on the brain circuits involved. Often this can be accomplished without the potential confounds of introducing a foreign substance into the brain or facing the many drawbacks of injecting a receptor blocker or agonist. Moreover, hydromineral balance is primarily regulated by a complex series of behavioral adjustments involving motivation, reward, social function, problem solving, and risk assessment. Given the diversity and far-reaching implications of these areas of research, there exists a translational potential for basic science understanding targeted at further revealing the mechanistic relationships between homeostatic and stress-responsive systems.

## Conflict of Interest Statement

The authors declare that the research was conducted in the absence of any commercial or financial relationships that could be construed as a potential conflict of interest.

## Footnotes

^1^http://mouse.brain-map.org/static/brainexplorer
